# Vagal Paraganglioma Presenting as a Neck Mass Associated with Cough on Palpation

**DOI:** 10.1155/2017/7603814

**Published:** 2017-06-22

**Authors:** Richard Heyes, Nizar Taki, Miriam A. O'Leary

**Affiliations:** Department of Otolaryngology, Head and Neck Surgery, Tufts Medical Center, 800 Washington St, Boston, MA 02111, USA

## Abstract

A 70-year-old female presented with a neck mass and sporadic dry cough, often leading to fits of coughing severe enough to cause vomiting. The patient reported that touching the mass triggered the cough. On examination, a 2.5 cm right-sided level two neck mass deep to the sternocleidomastoid was present. Palpation of the mass immediately triggered coughing. Cross-sectional imaging proposed vagal paraganglioma as the chief differential, which was confirmed following surgical excision. The patient reported complete resolution of her severe dry cough after surgery. Vagal paragangliomas are rare neuroendocrine tumors arising from the neural crest-derived paraganglionic tissue surrounding the vagus nerve, typically presenting as a neck mass associated with hoarseness or pulsatile tinnitus. To the best of our knowledge this is a unique description in the English literature. This case is presented to aid physicians should they encounter a neck mass associated with cough. Vagal paraganglioma, although rare, should be part of the differential in such a presentation.

## 1. Introduction

Vagal paragangliomas (VPs) are rare neuroendocrine tumors arising from the neural crest-derived paraganglionic tissue surrounding the vagus nerve. Paragangliomas (PGs) have classically been said to account for 0.6% of head and neck tumors, and, first described in 1935, VPs are the most rare subtype of head and neck PGs, contributing less than 5% of their total [1]. VPs typically present as a neck mass associated with hoarseness or pulsatile tinnitus [2]. PGs of the head and neck may undergo malignant transformation in 6% to 19% of cases; VPs are the most likely to become malignant and to metastasize [3]. There is potential for PGs to be metabolically active in secreting catecholamines, but this feature is present in only 2% of VP cases [3]. Management options include an observational approach, stereotactic radiosurgery, and surgical excision [1, 3]. Herein a unique case of vagal nerve paraganglioma is presented, where a neck mass was associated with severe fits of coughing upon palpation.

## 2. Case Report

A 70-year-old female presented with a 4-week history of a neck mass. She had suffered a concurrent sporadic dry cough unrelated to eating, talking, or any particular movement; fits of coughing would often be so severe that she would vomit. She reported that touching the neck mass would trigger the cough. The frequency and severity of the cough were diminishing her quality of life. She reported concomitant intermittent hoarseness. She denied dysphagia or velopharyngeal insufficiency. She had no family history of paragangliomas or other neuroendocrine tumors, or any relevant past medical history. On examination, a 2.5 cm right-sided level two neck mass deep to the sternocleidomastoid was present, which was discrete, mobile, and nonpulsatile. Palpation of the mass immediately triggered coughing. Flexible fiberoptic laryngoscopy demonstrated normal true vocal fold appearance and movement bilaterally, with prominent retropharyngeal carotid arteries the only finding of note. The remainder of the examination was unremarkable apart from a mildly hoarse voice.

**Figure 1:**
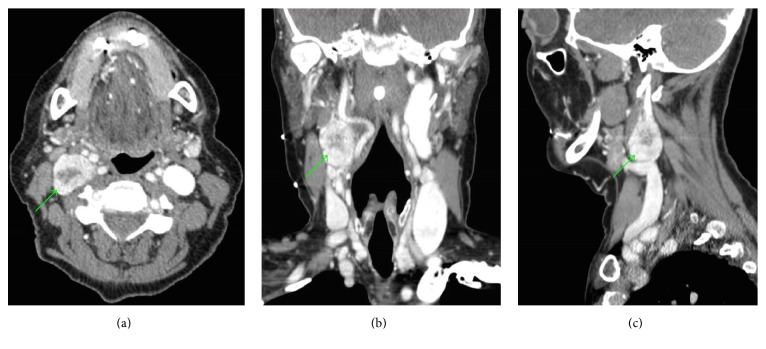
CT angiography of the neck with axial (a), coronal (b), and sagittal (c) sections of the heterogeneously enhanced irregular 2.4** × **2.2** × **2.2 cm mass within the right carotid sheath. The green arrows refer to the neck mass.

**Figure 2:**
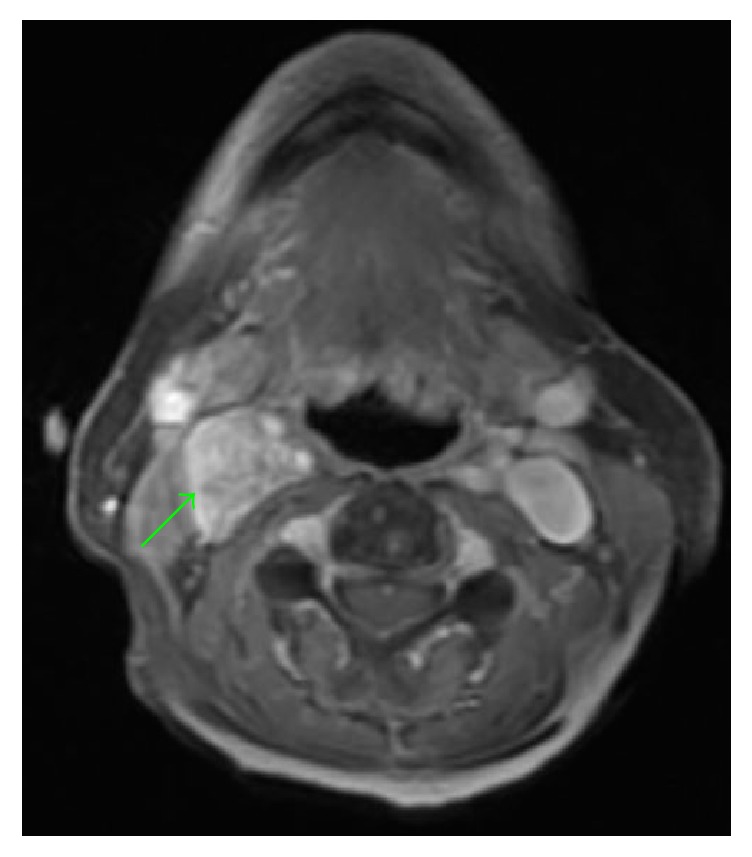
MRI, axial section, fast spoiled gradient echo with contrast. The green arrow refers to the neck mass.

**Figure 3:**
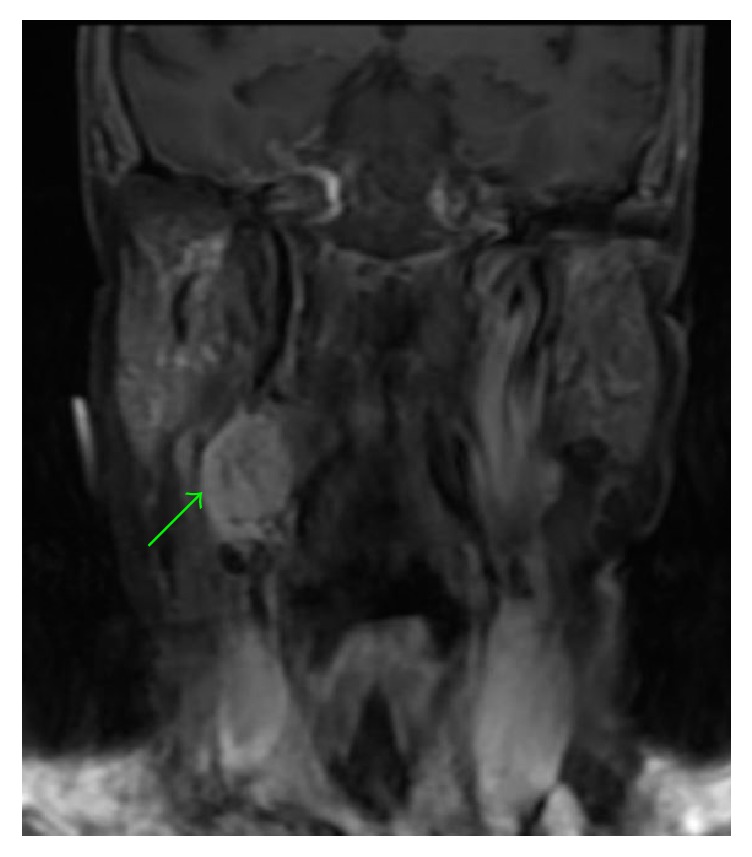
MRI, coronal section, T1-weighted with fat saturation and contrast. The green arrow refers to the neck mass.

A blood panel was normal and 24-hour urine was negative for catecholamines. CT angiography of the neck demonstrated a heterogeneously enhanced irregular 2.4 × 2.2 × 2.2 cm mass within the right carotid sheath along the lateral aspect of the proximal internal and external carotid arteries with minimal splaying of these arteries (Figures 1(a), 1(b), and 1(c)). The central area of the mass was of lower attenuation suggestive of necrosis. MRI displayed a 2.5 × 1.9 × 3 cm mass extending from the inferior margin of the right carotid to the level of the piriform sinuses within the carotid sheath which was intensely enhanced following contrast administration (Figures 2 and 3). Differential diagnoses included vagal paraganglioma, schwannoma, neurofibroma, or a necrotic lymph node. The CT and MR images were reviewed at our institution's head and neck radiology conference, with the consensus favoring a schwannoma over a paraganglioma due to the relatively homogenous appearance and lack of flow voids within the mass. To differentiate between these two pathologies, Indium-111-labelled octreotide scintigraphy was recommended. Octreotide is a somatostatin analogue which accumulates within paragangliomas due to a high density of somatostatin type 2 receptors on their cell surface; schwannomas do not uptake octreotide [4]. The mass was intensely avid on octreotide scan at both 4 and 24 hours with no additional visible masses. Therefore VP was now unequivocally the most likely pathology. The patient was counseled on management options. Due to the impact of the cough on her quality of life, she elected for surgical removal of the mass.

The mass was excised through a 7 cm incision made two finger-breadths below the mandible, overlying the sternocleidomastoid. Right perifacial and level II lymph nodes were resected to improve access to the mass. The mass was dissected free from the internal and external carotid arteries and the internal jugular vein. The vagal nerve was intimately involved with the mass, thus requiring suture ligation and removal with the mass. The spinal accessory and hypoglossal nerves were identified and preserved. The pathology report described a completely excised encapsulated 3.5 × 2 × 1.5 cm tumor associated with a nerve segment and ganglion. The central 10% of the tumor showed tumor necrosis. Immunohistochemical staining for SDHB was positive, with intratumoral heterogeneity. Positive immunostaining for SDHB suggests that the patient does not have a germline mutation that caused her paraganglioma; however, the significance of intratumoral heterogeneity is unclear, and therefore genetic counseling and the option of genetic testing were recommended. All dissected lymph nodes were negative for malignancy.

On postoperative evaluation in the office, the patient reported complete resolution of her severe dry cough after surgery. She was extremely pleased with this result. Her glottic closure and vocal quality remained good in the early postoperative period; therefore she chose to defer intervention for her vocal fold paralysis, with the understanding that her hoarseness would worsen over time as vocal fold atrophy occurred. She had no dysphagia after surgery and continued to tolerate a regular diet without difficulty. Genetic counseling and the option of genetic testing were discussed with the patient, based on the suggestion of the interpreting pathologist, which the patient declined. She elected to pursue further follow-up visits and future voice rehabilitation with a local otolaryngologist in her community.

## 3. Discussion

Paragangliomas arise from the paraganglia, which are small groups of neuroendocrine cells stemming from autonomic nervous system ganglia. Usually slow growing and benign, tumors of the paraganglia are most common in the adrenal medulla (pheochromocytoma), with 85% of extra-adrenal PGs in the abdomen, 12% in the thorax, and 3% in the head and neck [1]. Four genetic PG syndromes have been described, all with autosomal dominant transmission. Germline mutations are known in three, all in the gene complex encoding succinate dehydrogenase (SDH): with subunits SDHD, SDHC, or SDHB mutated, any of which predisposes PGs [3]. SDH is a mitochondrial enzyme complex with an important role in oxidative phosphorylation and intracellular oxygen sensing and signaling. It is thought that SDH mutations cause dysregulation of hypoxia-induced factors, thereby yielding a cellular response mimicking that of hypoxia, which is known to cause carotid paraganglial hypertrophy [5]. However, more than half of head and neck PGs are sporadic without an identifiable genetic cause.

Surgical excision is the classical treatment of choice for most VPs; however contemporary management is evolving toward more conservative measures due to the high associated morbidity [1]. VP resection almost always requires vagus nerve sacrifice with resultant speech, swallow, and sensory deficits [1, 3, 6]. Observation with serial imaging has been successfully employed, with the majority of lesions remaining radiologically stable, although neuropathy progression has occurred in a third of the cases [1, 3, 6]. Observation is especially valuable for asymptomatic older patients [1]. Stereotactic radiosurgery is a proven treatment option for VPs < 3 cm in maximum dimension and should be offered as a potential first-line treatment [1].

In the presented case, the severity of symptoms resulted in the management undertaken. A report exists describing a patient with a cough associated with VP; however this was a mild chronic cough, complicated by gastroesophageal reflux, and cough was not triggered by palpation of the mass [7]. Bouts of syncopal coughing associated with an intracranial jugular foramen paraganglioma have been reported, with transient cough-related increases in intracranial pressure as the proposed mechanism [8].

## 4. Conclusion

A neck mass associated with cough on its palpation has previously been described as pathognomonic for vagal nerve schwannoma [9], yet an alternate diagnosis was reached in this case. To the best of our knowledge this is the first description in the English literature of a VP presenting as a neck mass associated with a severe cough that could be triggered by palpation of the mass. This case is presented to aid physicians should they encounter a neck mass associated with cough. Vagal paraganglioma, although rare, should be part of the differential in such a presentation.
